# Laser synthesized TiO_2_-based nanoparticles and their efficiency in the photocatalytic degradation of linear carboxylic acids

**DOI:** 10.1080/14686996.2017.1379858

**Published:** 2017-10-25

**Authors:** Sarah Bouhadoun, Chantal Guillard, Sébastien Sorgues, Alexandre Hérissan, Christophe Colbeau-Justin, Frederic Dapozze, Aurélie Habert, Vincent Maurel, Nathalie Herlin-Boime

**Affiliations:** ^a^ NIMBE, CEA, CNRS, Université Paris Saclay, Gif sur Yvette, France; ^b^ Institut de recherche sur la catalyse et l’environnement, IRCELYON, CNRS-University of Lyon, Villeurbanne, France; ^c^ Univ. Grenoble Alpes, CEA, CNRS, INAC, SyMMES, Grenoble, France; ^d^ Laboratoire de chimie physique, UMR 8000-CNRS, Université Paris Saclay, Orsay, France

**Keywords:** Titanium dioxide nanoparticles, gold nanoparticles, co-modification with nitrogen, time resolved microwave conductivity, photocatalysis, Electronic Paramagnetic resonance, 60 New topics / Others, 205 Catalyst / Photocatalyst / Photosynthesis, 502 Electron spectroscopy, 305 Plasma / Laser processing, Gas phase Synthesis, 600 Time Resolved Microwave Conductivity

## Abstract

Titanium dioxide nanoparticles were synthesized by laser pyrolysis, their surface and electronic properties were modified by gold and/or nitrogen. These materials were characterized by different techniques like X-ray diffraction (XRD), X-ray photoelectron spectroscopy (XPS) and electron paramagnetic resonance (EPR). Time resolved conductivity (TRMC) was used to study the charge separation of electron/hole pairs. Altogether (XPS, EPR, TRMC), the physicochemical characterizations are well correlated with chemical photoactivity of the different samples. Their photocatalytic activity was evaluated for the degradation of linear carboxylic acids (C2-C3) under UV and visible illumination. The decomposition rate of acids was measured, it shows that the modification with gold increases the photoactivity while the presence of nitrogen slows down the process. Such observations are in good agreement with evolution of TRMC signals. A degradation pathway has been determined by identification of intermediate products by chromatography and EPR, results show different intermediate species. In particular EPR confirms the presence of NO^2−^ paramagnetic centers and shows two novel N centered paramagnetic centers. A decrease of the degradation rate is observed with increase of carboxylic acid chain length.

## Graphical abstract

**Figure:**
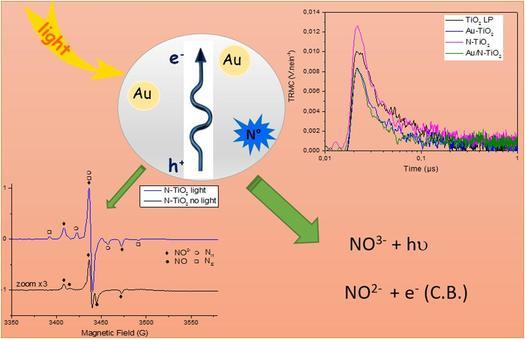

## 1. Introduction

Many studies deal with the fabrication and characterization of the photocatalytic activity of various materials exhibiting efficiency both in the visible and UV range [1–3]. In particular, materials based on TiO_2_ are intensively studied due to their high activity under UV illumination. The main drawbacks are high recombination rate and lack of activity under visible light. Improving photocatalytic efficiency under visible illumination would allow taking advantage of solar irradiation that is a free, abundant and non-polluting energy [4–7]. Different ways to enhance the visible photoactivity are doping by hetero-elements (N, S, F,…) or introducing metallic structures (Au, Ag…) with an absorption in the visible range and/or able to improve the charge separation [8–14]. In this context, we have previously demonstrated that the laser pyrolysis is an efficient method for the one step synthesis of TiO_2_-based nanomaterials (Au/TiO_2_, N-TiO_2_ and co-modified Au/N-TiO_2_) [15]. Under UV illumination, these samples were very efficient for the degradation of formic acid by comparison with commercial TiO_2_ P25. In particular gold modification of titanium dioxide enhances transfer of electrons. Interestingly, a significant activity was observed under visible illumination for our both materials containing nitrogen [15].

In parallel to materials development, advanced characterization methods are used to study their characteristics (crystallization in preferred directions, surface states,…) and correlate them to photocatalytic properties [14,16,17]. Previous EPR studies have shown the existence of paramagnetic nitrogen species interacting with the TiO_2_ lattice. These paramagnetic species are involved in the photo-induced electron transfer from the solid to an electron scavenger such as molecular oxygen [3,17,18]. Time Resolved Microwave Conductivity (TRMC) was used to demonstrate that Au nanoparticles can act as electron scavengers retarding recombination in Au-TiO_2_ samples [14,19] In this paper, we apply TRMC to characterize the ability of our sample to study charge carrier dynamics under UV or visible illumination. Based on TRMC and EPR results giving information on the presence of paramagnetic centers, we establish a qualitative classification in our family of samples towards their ability to separate the charges. Indeed, it is well known that charge separation is of major importance in the efficiency of photocatalyst materials [20]. In combination with other methods (such as X-ray photoelectron spectroscopy (XPS), EPR and photoluminescence [15]), we use the information obtained from TRMC to correlate the physicochemical properties to photocatalytic activity, i.e. ability of these samples to decompose various linear (C1 – C3) acids under UV or visible light. Indeed, by using different excitation wavelengths, TRMC gives information on the number of exciton created under UV or visible illumination for one type of sample and therefore predict their relative photocatalytic efficiency under both types of illumination.

## 2. Experimental details

### 2.1. Chemicals

Titanium tetraisopropoxide (TTIP, 97% purity) and hydrogen tetrachloroaurate (III) HAuCl_4_•3H_2_O were respectively used as titanium source for synthesis of TiO_2_ nanoparticles, as gold source. Ammoniac (NH_3_) gas was supplied by Messer. These reactants and the carboxylic acids CH_3_COOH, and CH_3_CH_2_COOH were obtained from Sigma-Aldrich and used without further purification. Commercial reference TiO_2_ P25 was obtained from Evonik.

### 2.2. Synthesis

Modified nanoparticles (NPs) were synthesized through laser pyrolysis method by using HAuCl_4_•3H_2_O dissolved in TTIP and NH_3_ as precursors. Briefly, this method is based on the interaction between a flow of precursors and a high power CO_2_ laser beam. The reactants are heated and dissociated with appearance of a flame. Homogeneous nucleation occurs; nanoparticles grow in the hot zone and are collected downstream on metallic filters. More details were reported in our previous work [15].

The obtained powders were annealed at 400 °C under air flow (650 cm^3^ min^−1^) in an oven (Pyrox) during 3 h to remove the residual carbon due to the decomposition of reactants (TTIP). All the results presented here concern annealed powders.

### 2.3. Characterization methods

The identification of crystallite phase of the synthesized photocatalysts was carried out by X-ray Diffraction measurements (XRD, Siemens D5000 diffractometer with Cu-Kα radiation (λ = 1.54184 A°)). The 2*θ* angle ranged from 20° to 80° with a step size of 0.04° and a counting time of 7s/step. The average TiO_2_ crystallite size of the sample was calculated from the Scherrer equation. The chemical environment of nitrogen atoms in TiO_2_ was deduced by X-ray photoelectron spectra (XPS) (Kratos Analytical Axis Ultra DLD spectrometer, Al Kα X-ray) on the powders.

The charge-carrier lifetimes in TiO_2_ after UV and visible illumination were determined by TRMC [21,22] .The TRMC technique is based on the measurement of the change of the microwave power reflected by a sample, Δ*P*(*t*), induced by its laser pulsed illumination. The relative difference Δ*P*(*t*)*/P* can be correlated, for small perturbations of conductivity, to the difference of the conductivity Δσ(t) considering the following equation:



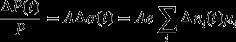



where Δn_i_(t) is the number of excess charge-carriers *i* at time *t* and *μ*_*i*_ their mobility. The sensitivity factor *A* is independent of time, but depends on different factors such as the microwave frequency or the dielectric constant. Considering that the trapped species have a small mobility, which can be neglected, Δn_i_ is reduced to mobile electrons in the conduction band and holes in the valence band. And in the specific case of TiO_2_, the TRMC signal can be attributed to electrons because their mobility is much larger than that of the holes [23].

The incident microwaves were generated by a Gunn diode of the K_α_ band at 30 GHz. Pulsed light source was an optical parametric oscillator (OPO, EKSPLA, NT342B) tunable from 225 to 2000 nm. It delivers 8 ns pulses (full width at half maximum) with a frequency of 10 Hz. The light energy densities received by the sample were respectively 1.2, 3.4, and 7.0 mJ∙cm^−2^ at 355, 410, and 450 nm. The main data provided by TRMC are given by the maximum value of the signal (I_max_), which reflects the number of the excess charge-carriers created by the pulse and the decay of the signal I(t), which is due to the decrease of the excess electrons controlled by recombination and trapping.

Low-temperature continuous-wave EPR spectra were recorded with a Bruker EMX spectrometer operating at X-band (9.65 GHz) frequency and equipped with an ER-4116 dual mode cavity and an Oxford Instruments ESR-900 flow cryostat. For the study of photoirradiated dry photocatalyst powders, *in situ* irradiation of samples in the EPR cavity was achieved using a fibered Schott KL1500 halogen UV-visible lamp. For the study of degradation of carboxylic acids (0.1 M) in aqueous dispersion of photocatalyst (4 g L^−1^), the samples were frozen in liquid nitrogen and irradiated in a Rayonet UVA photoreactor (8 mW cm^−2^.) for 30 min at T = 77 K in a quartz cold finger, then transferred into the EPR spectrometer at T = 60 K.

### 2.4. Photocatalytic experiments

The photocatalytic efficiency of samples was evaluated by following the degradation of linear acids (1086 μmol L^−1^) in water and 181 μmol L^−1^ corresponding to a similar concentration of carbon. The photocatalytic tests were carried out in a Pyrex photoreactor. For UV irradiation, a mercury lamp Phillips HPK 125 W with optical filters 7.6 and 0.52 (Corning) were employed to obtain an emission peak centered at λ = 365 nm. The radiant flux at 4.2 mW cm^−2^ (7.7 10^15^ photons s^−1^ cm^−^²) was measured using a VLX-3 W radiometer with 365 nm sensor. For visible light illumination in the region (400–800 nm), LED lamp was used; the photon flux was measured around 85 10^−3^ μmol s^−1^. Both lamps were placed at the bottom of the reactor; the illuminated area was 12.5 cm². Circulating water between lamp and reactor maintain the solution temperature at 20 °C. Before irradiation, the system with TiO_2_ concentration of 1 g L^−1^ was always stirred in the dark for 30 min, to reach the adsorption-desorption equilibrium of carboxylic acids on surfaces.

At regular time intervals of irradiation, aliquots of each acid suspension was collected, filtered using 0.45 μm Millipore filters before analysis. The concentrations of acids remaining after adsorption and during the photocatalytic degradation process were determined by high-performance liquid chromatography (HPLC) analysis. VARIAN Prostar HPLC apparatus equipped with a single-wavelength UV-vis detector and a 300 mm x 7.8 mm carbohydrate analysis column (COREGEL-87H3) was used. The mobile phase was H_2_SO_4_ solution (5 10^−3^ mol L^−1^) and the flow rate was fixed at 0.7 cm^3^ min^−1^. The detection wavelength was set at 210 nm.

## 3. Results and discussion

Based on our previous synthesis and photocatalytic results [15], four samples were selected for this study. The samples are TiO_2_ LP, Au/TiO_2_, N-TiO_2_ and co-modified Au/N-TiO_2_ and their main characteristics are presented in Table S1. The samples were synthesized by laser pyrolysis in similar conditions, except the choice of the reactive mixture injected in the laser beam. The TiO_2_ LP was chosen as a reference and for comparison with TiO_2_ P25, Au/TiO_2_ sample was selected to demonstrate the effect of the metallic additive. N-TiO_2_ was chosen because its visible activity is well known [24,25]. In addition the co-modified Au/N-TiO_2_ sample exhibiting significant activity in the visible while maintaining good activity under UV illumination is part of the study [26,27].

### 3.1. XRD and XPS characterization of materials

XRD patterns of pure and modified TiO_2_ powders [15] show a major anatase crystallite phase with less than 5% of rutile (Figure S1), this ratio was estimated from the peaks located at 2*θ* = 25.32°(anatase) and at 2*θ* = 27.45° (rutile). The anatase crystallite size was evaluated using the Scherrer equation. The mean crystallite size deduced from XRD measurements is in the range 7–9 nm for all these samples, in quite good agreement with the grain size deduced from transmission electron microscopy measurements (Table S1).

**Figure 1.**
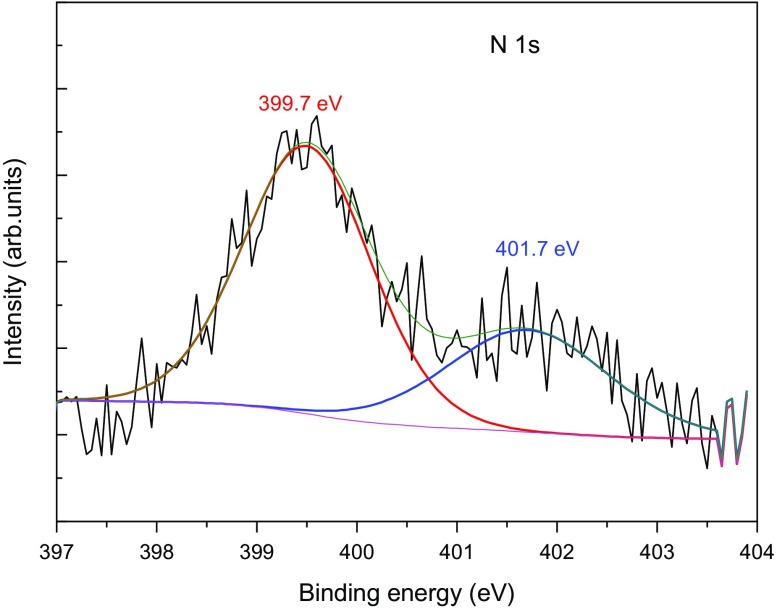
XPS spectrum of N-TiO_2_ powder.

The XPS spectra of all nitrogeN-doped and gold modified TiO_2_ have been recorded. The spectra corresponding to Ti 2p and O 1s levels are shown in Figure S2. As usually observed for the Ti 2p spectra, two peaks are present at 458.5 eV and 464.3 eV assigned to Ti 2p_3/2_ and Ti 2p_1/2_ in agreement with the presence of Ti^4+^ in a TiO_2_ environment. The O 1s binding energies of all powders were located at energies slightly higher than 530 eV, attributed to O^2−^ in the TiO_2_ lattice. Let us note the Au could not be detected, probably due to the low content below the detectivity level of XPS. In the N-doped samples (N-TiO_2_ and Au/N-TiO_2_), the N local environment has also been studied. The spectra and fitting curves are presented in Figure 1 (The spectrum of Au/N-TiO2 sample is very similar and is therefore not shown here). Two peaks are present; their binding energies at 399.7 and 401.7 eV as already observed in the literature and are assigned to O-Ti-N bonds (interstitial N) [18,28,29] and to molecularly adsorbed N species like NO or NO_2_ molecules on the particle surface [30], respectively. It is therefore important to note here that a significant part of our nitrogen is located at the surface of nanoparticles.

### 3.2. *EPR study of charge carriers and paramagnetic species*

**Figure 2.**
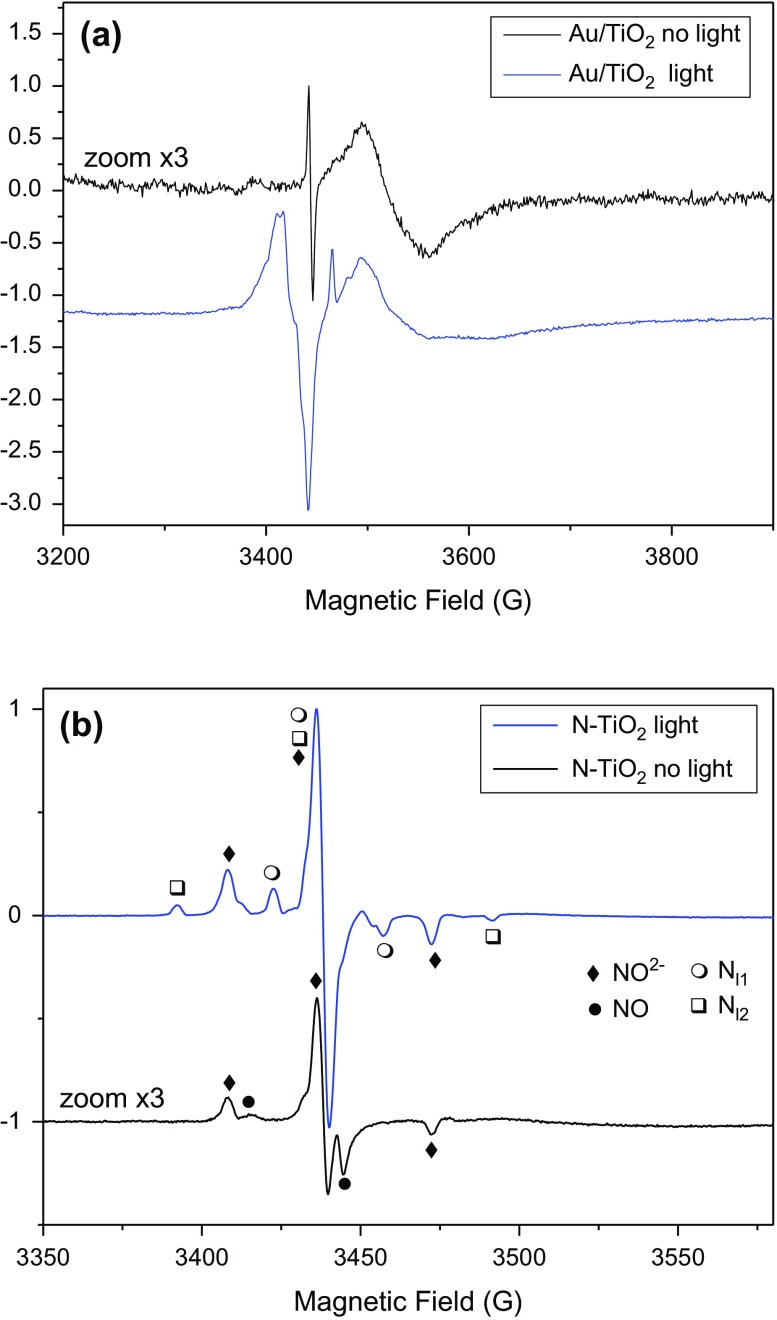
EPR spectra observed for dry photocatalysts powder without (dark curves) and with (blue curves) *in situ* UV-visible (halogen-lamp) irradiation at T = 60 K. (see exp. section for details). (a) Au modified sample, (b) N-doped sample.

N-doped and Au-modified samples (Au/TiO_2,_ N-TiO_2_ and Au/N-TiO_2_) were also studied by EPR at low temperature (60 K) in the dark as well as in the presence of UV-visible light irradiation (Figure 2).

Looking at the EPR spectrum recorded for Au/TiO_2_ samples, (Figure 2(a)), the signal in the dark is of weak intensity. It is composed of a broad part due to trapped electrons (Ti^3+^ in rutile and surface Ti^3+^, Table 1) and a sharp line at g = 2.002 tentatively attributed to bulk anion vacancy defects [31]. Such signals were previously reported in EPR studies of Au/TiO_2_ catalysts prepared by deposition/precipitation methods [31]. Under UV-visible light irradiation, this sample exhibits much more intense EPR signals due mostly to the presence of charge carriers. Trapped electrons including Ti^3+^ in rutile, Ti^3+^ in anatase and Ti^3+^ in surface sites are observed at g values lower than 2. Holes trapped in anatase and or rutile phases are also observed with signals at g = 2.021 and 2.018 (Table 1 and [32]). The remaining smaller features at g > 2 can be attributed to O_2_^−^ stabilized at anatase surface or Ti^4+^O^2−^Ti^4+^O^°−^ sites [31,32].

**Table 1.** EPR parameters of species observed for dry photocatalysts powders.

	g tensor			A_N_ tensor (G)			Reference
	(g_1_≥g_2_≥g_3_)			(A_1_,A_2_,A_3_) in g frame			
Bulk anion vacancy	2.002			–			Okumura et al*.* [31]
Ti^3+^ anatase	1.990	1.990	1.960	–			Kumar et al. [32]
Ti^3+^ rutile	1.970	1.970	1.944	–			Kumar et al*.* [32]
Surface Ti^3+^ at pH 10	1.945	1.945	1.885	–			Howe and Grätzel [39]
rutile TiO_2_ (Ti^4+^O^°−^Ti^4+^OH^−^)	2.019	2.014	2.002	–			Kumar et al*.*[32]
anatase TiO_2_ (Ti^4+^O^°−^Ti^4+^OH^−^)	2.016	2.012	2.002	–			Kumar et al*.* [32]
Ti^3+^ surface reported for C-doped	1.971	1.971	1.948	–			Li et al*.* [33]
NO°	2.001	1.998	1.927	<1	32.2	9.6	Livraghi et al*.*[34][35]
NO^2−^	2.005	2.004	2.003	2.3	4.4	32.2	Livraghi et al. [17]
N_l1_°	2.007	2.055	2.003	–	–	17.2	This work
N_l2_°	2.005	2.004	2.0023	–	–	49.5	This work

The EPR spectrum recorded for N-TiO_2_ (Figure 2(b)) in the dark is much more intense than the spectrum of Au/TiO_2_ in the dark, as also shown by the signal to noise ratio, and different species are present. By numerical simulation of the EPR spectrum (Figure S3), it was possible to identify four main species with important contributions in the EPR spectrum : surface Ti^3+^ with similar g tensor as reported for C-doped TiO_2_ [33], a second surface Ti^3+^ site similar to the one observed for Au/TiO_2_, NO° radicals [34,35] and a last paramagnetic center exhibiting nitrogen hyperfine splitting. This signal was attributed to a nitrogen center labeled as N_b_° in many papers in the literature [18,35,36], then attributed to interstitial or substitutional N atom in TiO_2_ [16] and finally identified as paramagnetic NO^2−^ species trapped in bulk TiO_2_ [17,37]. When the sample is irradiated *in situ* with halogen lamp, the spectrum changes drastically (Figure 2(b)). The contributions of Ti^3+^ in rutile, surface Ti^3+^ and NO° radical vanish. Signal from holes is no more observed. The signal due to NO^2−^ increases by a factor of five and two new paramagnetic centers exhibiting different N hyperfine coupling appear (named N_l1_° and N_l2_° in Table 1).

The increase of EPR signal attributed to NO^2−^ centers in N-TiO_2_ samples under visible or UV-visible irradiation has been already observed [37,38] and was rationalized by Barolo et al*.* [37]. These authors report the presence in the synthesized nanoparticles of a diamagnetic center (NO^3−^). This species is responsible of the visible absorption in N-TiO_2_ materials. It does not create [electron-hole] pairs in N-TiO_2_, but rather promote an electron from diamagnetic NO^3−^ intraband gap states to the conduction band, thus creating more paramagnetic NO^2−^ intraband gap states.



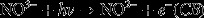



The produced electron is transferred onto the scavenger (O2) to form O^2°−^



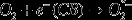



On the other hand to the best of our knowledge the two other paramagnetic centers exhibiting different N hyperfine coupling have not been described in the literature. They disappear when the sample is warmed up to room temperature and were observed only during or just after irradiation at low temperature, so they will be named N_l1_° and N_l2_° hereafter (the subscript ‘l’ stands for ‘light’).

The EPR spectrum recorded for Au/N-TiO_2_ (Figure S4) in the dark exhibits only NO^2−^ paramagnetic centers. No contribution from NO neither from Ti^3+^ was observed. Under UV-visible irradiation the EPR spectrum is comparable to this observed for N-TiO_2_ but approximately twice less intense: the same paramagnetic centers NO^2−^, N_l1_° and N_l2_° are observed under UV-vis irradiation.

Finally, by comparing the EPR spectra recorded with UV-visible irradiation of Au/TiO_2_, N-TiO_2_ and Au/N-TiO_2_ in exactly the same conditions, one can see that nitrogen doping induces drastic changes. UV-visible photoirradiated Au/TiO_2_ exhibits both EPR signals due to holes and electrons as observed in classical TiO_2_ samples. On the opposite, no EPR signal from holes nor photogenerated electrons could be observed in N-TiO_2_ and Au/N-TiO_2_. So according to these results N containing samples produce much less electron and holes than Au/TiO_2_ under UV-visible irradiation. Instead of producing holes, photoirradiation promotes mainly the formation of paramagnetic NO^2−^ species which may induce a less good reactivity for the photooxidation of carboxylic acids.

### 3.3. Charge carrier dynamics

The electronic properties of the samples were studied by TRMC technique considering both UV and visible regions. The excitation wavelengths were 355 and 450 nm. Let us note that such comparison of the samples is possible because all the samples were obtained by the same method in similar conditions

#### 3.3.1. UV excitation (355 nm)

**Figure 3.**
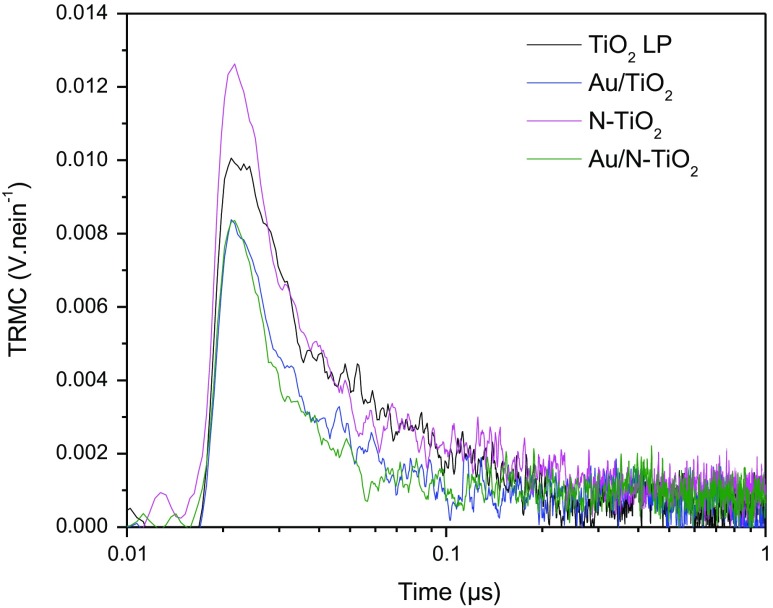
TRMC signal of pure and modified TiO_2_ obtained by irradiation at 355 nm.

Figure 3 presents the TRMC signals recorded for the different samples after UV excitation. In Table 2, column 2 gives the maximum value of the signal proportional to the number of excitons created under illumination while column 3 allows comparison of the short range decay behavior of the different samples. Column 4 presents the K_D_ adimensional parameter related to long-term lifetime of charge carriers (after 100 ns), higher K_D_ corresponds to faster decays of the TRMC signal. This parameter is obtained using the I = I_D_ × t^−kD^ expression as proposed in the paper of Meichtry et al*.* [40]*.* Figure 3 shows similar behavior for all the samples : a classical anatase signal achieving a maximum value at about 20–25 ns followed by an anatase long decay with recombination phenomena at short-time range (0 to 40 ns) and trapping phenomena at longer-time range (after 40 ns) and mainly noise after 100 ns [41,42]. The 0.18 and 0.19 k_D_ values obtained for samples containing gold show that there is almost no slow processes (after 100 ns) in these samples. Considering the TiO_2_ LP and N-TiO_2_ samples, the higher k_D_ values indicate the presence of phenomena occurring at long-time range such as decay of excess electrons [40] in good agreement with the higher number of electrons in theses samples (Figure 3).The influence of the incorporated gold can be also observed by comparing the signals measured on TiO_2_ and Au/TiO_2_ materials (Figure 3). First, at short-time range, gold acts like an impurity. It can be observed by the reduction of I_max_ and a lower value of I_40 ns_/I_max_ (0.34 compared to 0.27 in Table 2), corresponding to a faster decay of the electrons population in Au/TiO_2_ sample. This decay can be associated either to recombination phenomena or transfer of charges. In this case, photoluminescence experiments have already indicated an electronic transfer from TiO_2_ to gold, therefore the TRMC result can be safely attributed to the transfer of electrons to gold and is in good agreement with an improved photocatalytic activity of Au/TiO_2_ in comparison with TiO_2_ LP as already observed for the degradation of formic acid (FA). Indeed, the effect of Au and/or Cu metallic additives on P25 was studied by TRMC [19]. It induced a faster transfer of electrons to the surface correlated to an improved photocatalytic efficiency similar to our present work.

**Table 2.** TRMC parameters for pure and modified TiO_2_ obtained by irradiation at 355 nm.

Sample	I max (V.nein^−1^)*	I_max_ (mV)	I_40 ns_/I_max_	k_D_
TiO_2_ LP	0.010	69	0.34	0.67
Au/TiO_2_	0.008	55	0.27	0.19
N-TiO_2_	0.013	84	0.22	0.36
Au/N-TiO_2_	0.08	55	0.16	0.18

* V. nein^−1^ stands for Volt per nano-Einstein, i.e. mol of photons.

If we now turn to the N-doped sample, a first effect can be seen at short-time range. I_max_ is slightly higher for the N-doped compound (84 mV vs 69 mV for the TiO_2_ sample) indicating a better charge-carrier creation. However, like previously, this effect is accompanied by a faster loss of electrons (lower I_40 ns_/I_max_, 0.22 compare to 0.34) and no long-time range effects are detected: TRMC signals of pure and doped compounds are equal after 40 ns. In the N-TiO_2_ samples the faster loss of electrons can be related to the presence of nitrogen in surface states as shown by XPS (Figure 1). Indeed, such surface states are known to act as recombination centers where the electrons are lost. Therefore, the N doping induces recombination which is in good agreement with the less efficient photocatalytic activity (FA degradation) observed for this sample under UV illumination [15].

The compound modified with Au and N shows the lowest I_40 ns_/I_max_ value, i.e. the fastest electron loss (0.16 vs 0.34 for TiO_2_). Indeed, in this sample, both N and Au tend to decrease the electron population both by transfer to gold and charge recombination at the surface, explaining this observation. However, it is not possible to quantify the respective contribution of these effects and explain the efficiency of Au/N-TiO_2_ by comparison to TiO_2_.

Finally, these results tend to indicate that the most active sample is Au/TiO_2_, the less active N-TiO_2_ while TiO_2_ and Au/N-TiO_2_ are in between but their respective efficiency cannot be anticipated. Taking into account the very low Au amount in the powder in Au/N-TiO_2_, it could expected that the negative recombination effects due to N at the surface will be dominant, and explaining the lower photoactivity (degradation of FA) of Au/N-TiO_2_ compared to TiO_2_ [15].

#### 3.3.2. Visible excitation (450 nm)

**Figure 4.**
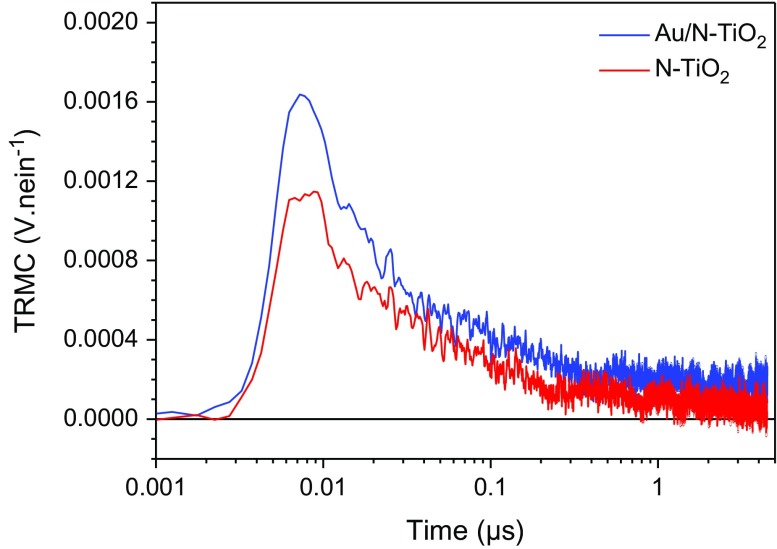
TRMC signal of N-TiO_2_ and Au/N-TiO_2_ obtained by irradiation at 450 nm.

Using the photons delivered by solar light in the visible range to increase the efficiency of photocatalytic processes remains a challenge, and N doping is known to induce some photocatalytic activity in the visible range [3,43] .In a previous study, we have shown that both N-TiO_2_ and Au/N-TiO_2_ samples are efficient for the degradation of FA under visible illumination. In the same way than previously, but using visible excitation, the creation and decay of exciton was measured by TRMC for our two N-doped samples exhibit some activity under visible range [15], Figure 4 shows the TRMC signals of N-doped and Au/N co-modified TiO_2_.

The main point of these data is the very low number of excitons created after visible excitation compared to UV excitation (0.001 vs 0.01 V.nein^−1^). The low number of excitons is easily correlated to the existing but weak efficiency observed for degradation of FA.

However, it clearly illustrates that the efficiency of our materials under visible excitation cannot be considered significant and this point will not be studied any more in the present paper. Moreover in good agreement with this low efficiency, monochromatic visible irradiation is known in literature to produce electron and not holes [37].

### 3.4. Comparison of EPR and TRMC under UV irradiation

UV-visible photoirradiated Au/TiO_2_ exhibits both EPR signals due to holes and electrons as observed in classical TiO_2_ samples. In agreement with a high number of electrons created under UV irradiation, the TRMC signal is of high intensity. Therefore, both techniques tend to indicate efficient surface activity.

The EPR signal observed with N-modified sample is mainly attributed to paramagnetic NO^2−^ species. In TRMC, although the charge carrier creation is the highest in N-TiO_2_ sample, the electrons are rapidly lost by recombination with surface traps. In fact, the NO^2−^ species seen by EPR under UV-visible irradiation (Figure 2), can act as traps for the electrons explaining the low efficiency of these samples.

### 3.5. Photocatalytic results

We have seen that, all our samples are significantly more active that TiO_2_ P25 when degradation of FA is considered [15]. In view of addressing more realistic pollutants, our interest was to understand if this result could be extended to more complex reactants while remaining in the same family (i.e. carboxylic acids). Therefore, the degradation of carboxylic acids with more carbon atoms (up to 3) was studied.

#### 3.5.1. Adsorption studies

Reaction at the surface of nanoparticles is a key mechanism in photocatalysis, it is therefore fundamental to investigate the evolution of the adsorption in the dark of the different reactants on the different materials. The adsorption results are presented in Table 3. It shows that for Laser synthesized powders, adsorption is significantly higher for FA compared to other acids. The difference is not so large for the TiO_2_ P25 reference sample. Table 4 shows pKa and pH of each acid (pH was measured in solution without nanoparticles). Based on these data the ratio of ionized [A^−^] to neutral [AH] species is much higher for FA than for acetic or propionic acid. It can explain the very different adsorption of FA compared to acetic and propionic acids (257 μmol g^−1^ compared 35 or 66 μmol g^−1^ for example in TiO_2_ LP). These results are also in agreement with the study by Serpone et al*.* [44] relating the adsorption behavior on TiO_2_ to the degree of ionization of acids. Indeed, the lower the p*K*_a_, the highest is the degree of ionization thereby favoring adsorption. Serpone et al*.* also predict a decrease of activity with the increment of the length of the carbon chain.

**Table 3.** Adsorbed quantities of carboxylic acids at equilibrium under dark for the different reactants and catalysts (μmol g^−1^).

Catalyst	TiO_2_ P25	TiO_2_ LP	Au/TiO_2_	N-TiO_2_	Au/N-TiO_2_
Formic acid	87	257	158	168	176
Acetic acid	60	35	70	35	35
Propionic acid	116	66	53	75	135

**Table 4.** Pk_a_ and pH of carboxylic acids (C1, C2, and C3).

Carboxylic acid	Formic acid	Acetic acid	Propionic acid
pK_a_	3.75	4.75	4.87
pH	3.3	3.7	3.8
A^-^/AH	0.39	0.090	0.085

#### 3.5.2. Photocatalytic degradation

**Figure 5.**
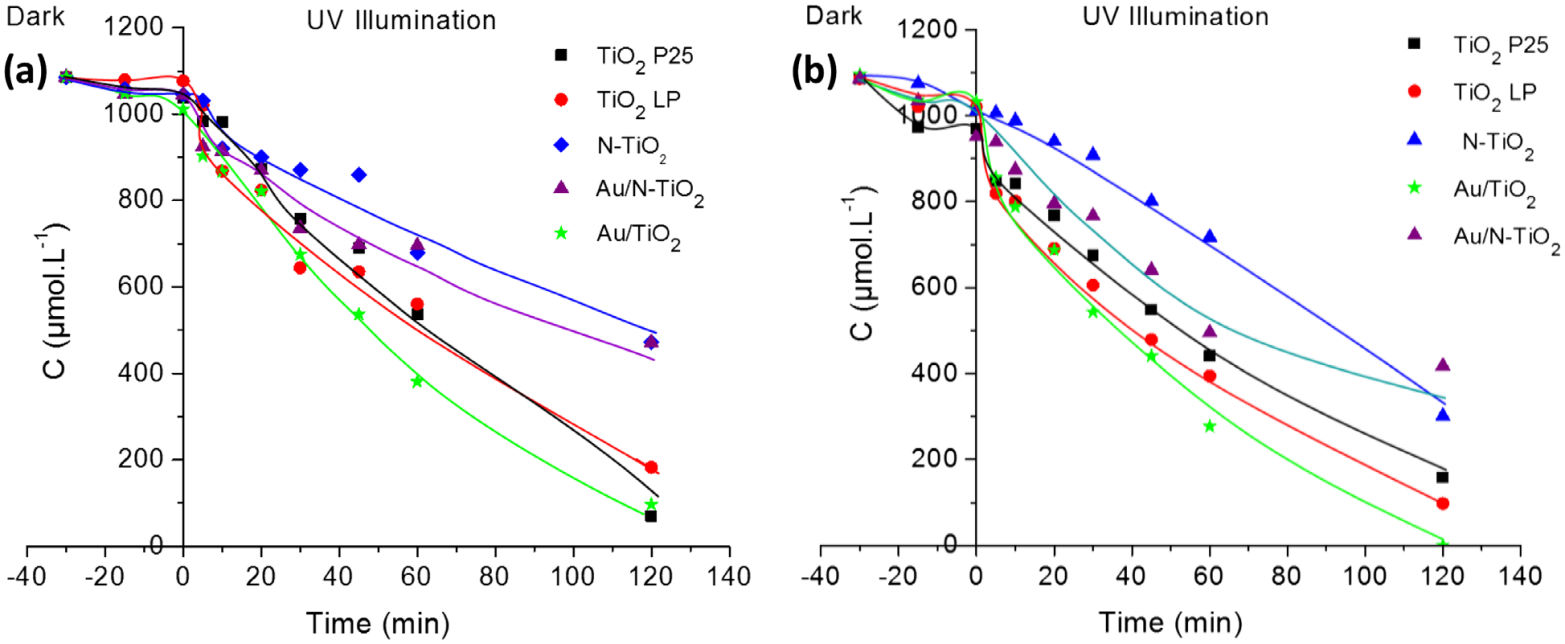
Photocatalytic decomposition of acetic acid (a) and propionic acid (b) under UV illumination for pure and modified TiO_2_ samples (the lines are only guides for the eyes).

**Figure 6.**
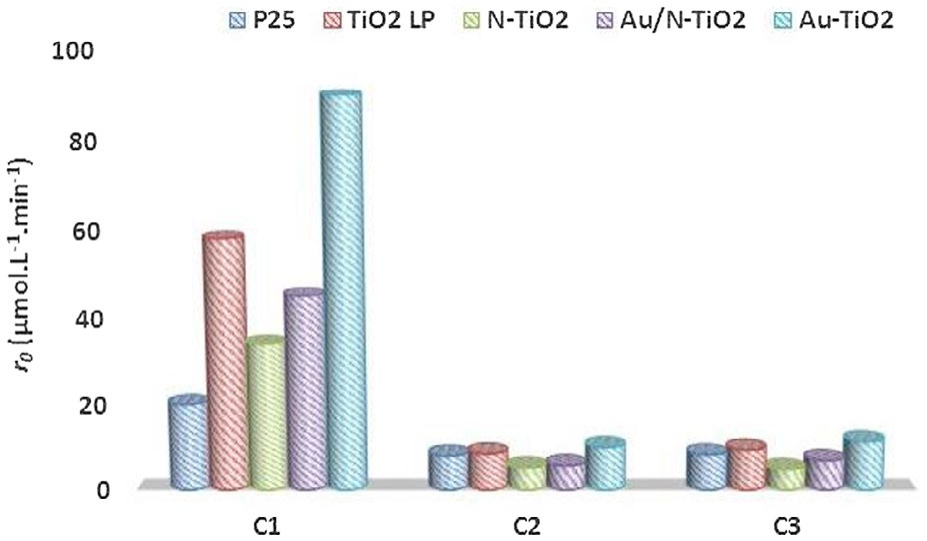
Evolution of the initial degradation rate (*r*_0_) as function of carbon chain**.**

Figure 5 presents the evolution of the concentration of C2 and C3 acids under UV irradiation in presence of modified TiO_2_ samples. In both cases, similar tendencies can be observed; the curves show that according to their behavior, the powders can be divided into two groups. The first group is composed of N-doped TiO_2_ samples; they are less active than TiO_2_ LP. TRMC, XPS and EPR results have shown the presence of nitrogen species that could act as recombination center. Therefore, the lower photocatalytic result is in good agreement with behavior expected from characterization. The second group is composed of P25, TiO_2_ LP and Au/TiO_2_ powders presenting a similar photoactivity. As in our previous study, the modification with Au has a positive effect on the photocatalytic efficiency: (Au/N-TiO_2_ and Au/TiO_2_ are more active than N-TiO_2_ and TiO_2_ LP) [15]. Finally, the photocatalytic activity follows the order: Au/TiO_2_ > TiO_2_ LP ≈ P25 > Au/N-TiO_2_ > N-TiO_2_ (Figure 6).

In comparison with our previous results on the degradation of formic acid under UV [15], kinetic decomposition of acetic acid (AA) and propionic acid (PA) is slower as illustrated in Figure 6. The kinetics of degradation of AA and PA are quite comparable, this seems correlated to adsorption very similar for most samples when these reactants are considered. In the same way, adsorption is significantly smaller for these two reactants compared to FA and the kinetics of degradation are slower, illustrating again the importance of surface reaction in the photocatalytic process. Moreover, the disappearance rates of FA, AA and PA molecules are correlated to the rate constants of OH^•^ attack radicals which are 1.4 10^8^, 1.six 10^7^ and five.7 10^6^ L.mol^−1^.s^−1,^ respectively [45].

### 3.6. Intermediate species and mechanism

One interest of using longer carbon chain corresponding to smaller degradation rates is the possibility to observe intermediate species giving information on the mechanisms involved in the degradation processes. Considering both formic and acetic acids, no peak could observed in the chromatograms (HPLC) at the end of the degradation process. Therefore, EPR analyses were carried out to obtain further insight into the nature of intermediates species and to characterize the local environment of the paramagnetic species.

**Figure 7.**
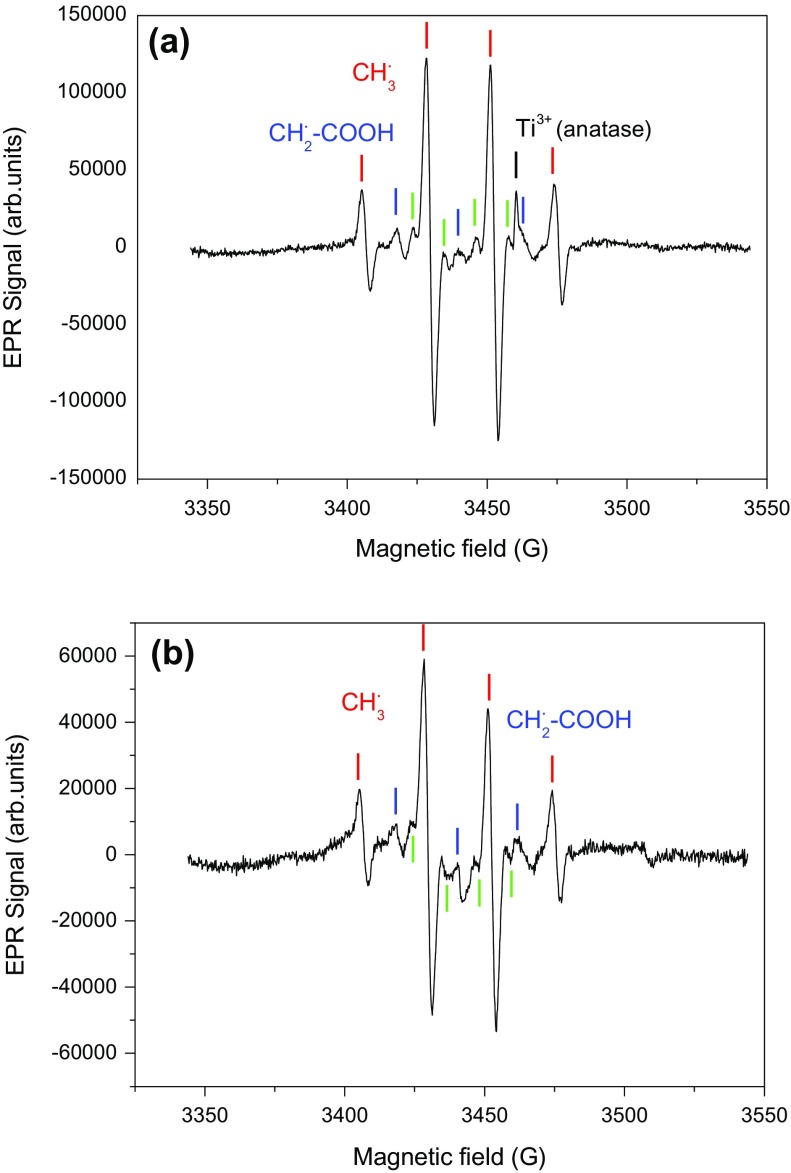
EPR spectra of (a) Au/TiO_2_ and (b) N-TiO_2_ in presence of acetic acid recorded at 77 K, and irradiated at λ = 365 nm.

Figure 7 shows the EPR spectra obtained with Au/TiO_2_ and N-TiO_2_ samples in the presence of acetic acid, recorded at 60 K and previously irradiated in a quartz cold finger at 365 nm and 77 K. Two intermediates were well identified, they can be assigned to methyl radical ^◦^CH_3_ with a proton hyperfine interaction (a_H,CH3_ = 22.9G), and to carboxymethyl radical ^◦^CH_2_-COOH (a_H,CH2_ = 21.1G). Four other narrow EPR lines (green labels in Figure 7) were observed and could be rationalized by a free radical containing two non-equivalent protons (a_H1_ = 23.2 G, a_H2_ = 11.4 G). To the best of our knowledge this last species was not reported to date in the literature of carboxylic acid photooxidation by TiO_2_, and no satisfying hypothesis could be found yet to rationalize it.

The observation of methyl and carboxymethyl radicals is consistent with the results reported by Kaise et al*.* [46] who explain their formation by the attack of photogenerated hole of TiO_2_ and then enter in the steps of photo-Kolbe reaction (Equation (1)). The same radicals were found by Nosaka et al*.* [47]. The formation of methyl radical is easily explained by the reaction of AA with a hole at the surface of the particle following reaction (1).


(1)
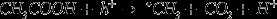



The resulting methyl radical can in turn react with AA to form carboxymethyl radical


(2)
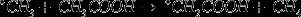



Another possible reaction can generate the carboxymethyl radical (Equation(3)) [48]


(3)
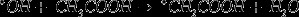



These radicals can follow reaction path with O_2_ to product alcohol, then aldehyde and finally CO_2_ in the case of ^∘^ *CH*_3_ or glycolic acid, glyoxylic acid, formaldehyde and then CO_2_ in the case of ^∘^ *CH*_2_ *COOH* [48].

**Figure 8.**
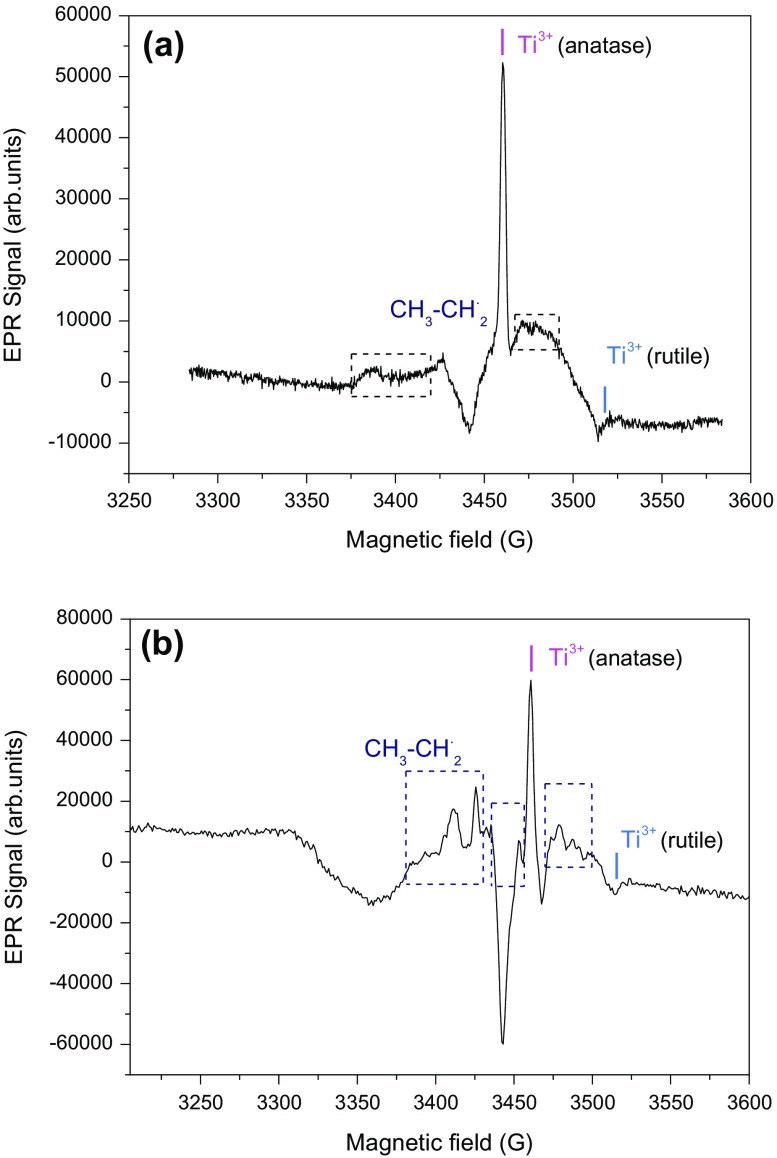
EPR spectra of (a) Au/TiO_2_ and (b) N-TiO_2_ in presence of propionic acid recorded at 77 K, and irradiated at λ = 365 nm.


During propionic acid photodegradation, only acetic acid was detected by HPLC. In this case, again EPR was used to detect eventual intermediates. Figure 8 shows the EPR spectra of Au/TiO_2_ and N-TiO_2_ samples in presence of propionic acid, recorded at 77 K and irradiated at 365 nm. Both of spectra show the typical signals corresponding to electron (Ti^3+^ species) in anatase and rutile environment [32]. Let us note that in this case the narrow Ti^3+^ signal is easily seen by contrast to the broader features observed in the spectrum. Some of these features could be attributed to ^∘^ *CH*_2_ – *CH*_3_ radicals by comparison with observation by Shkrob and Chemerisov [49] and appeared more clearly in the experiment performed with N-TiO_2_. Similarly to the observation by Shkrob and Chemerisov, the hyperfine splitting of ^∘^ *CH*_2_ – *CH*_3_ free radicals are not well resolved due to their anisotropy. Sakata et al. [50] proposed reaction path that explain the photocatalytic degradation of propionic acid (Equations (4-7)).

(4)
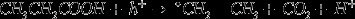




(5)
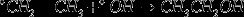

(6)
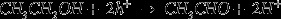

(7)
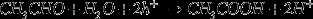



After these cascade reactions, the final product (acetic acid) will undergo the same reaction which was seen previously.

Finally, when HPLC could not give information (acetic acid), EPR study provides us the detection of intermediate species in the degradation mechanism in good agreement with the literature [47,49]. Considering propionic acid, EPR radicals support the presence of species identified by HPLC and confirm results of the literature [50].

## 4. Conclusions

The surface modification of titania by gold and/or nitrogen was successfully performed by using laser pyrolysis method. The as-synthesized materials show higher activity compared to TiO_2_ P25 under UV illumination for degradation of model organic pollutants (formic, acetic and propionic acids). However, co-modification of laser synthesized TiO_2_ with N and Au induces a decrease of the degradation rate of carboxylic acids in comparison with single modification by Au. TRMC results showed that under irradiation with UV light, the modification by Au improves photocatalytic efficiency of TiO_2_ due to more efficient transfer of electrons to the surface. On the contrary, presence of N causes recombination of charges, and thus gives rise to lower photoactivity in both Au/N-TiO_2_ and N-TiO_2_ samples. EPR investigations confirmed the presence of two radical species in N-TiO_2_ and Au/N-TiO_2_ samples. That result has never been observed with N-TiO_2_ synthesized by other methods, it seems to be specific to nanoparticles obtained by laser pyrolysis. As a complement, the intermediate species detected by EPR and HPLC allow proposing a reaction mechanism involved in the photo-oxidation of aliphatic organic acids. In order to improve photo-efficiency, it would be interesting to increase the size of gold nanoparticles and take advantage of surface plasmon resonance favoring activity in the visible range. In the same way, a better control of N doping favoring interstitial or substitutional doping would allow decreasing recombination of charges.

## Disclosure statement

No potential conflict of interest was reported by the authors.

## Supplemental data

The supplemental material for this paper is available online at https://doi.org/10.1080/14686996.2017.1379858

## Supplementary Material

sup_data_200617.docx
