# Supplementary material for: Laser synthesized TiO2-based nanoparticles and their efficiency in the photocatalytic degradation of linear carboxylic acids
Source: Sci Technol Adv Mater. 2017 Oct 25;18(1):805–15. doi: 10.1080/14686996.2017.1379858 (PMC5678285; doi:10.1080/14686996.2017.1379858)
Supplement: sup_data_200617.docx [file TSTA_A_1379858_SM9520.docx]

**Laser synthesized TiO_2_ based nanoparticles and their efficiency in the photocatalytic degradation of linear carboxylic acids**

Sarah Bouhadoun^a^, Chantal Guillard^b^, Sébastien Sorgues^d^, Alexandre Hérissan^d^, Christophe Colbeau-Justin^d^, Frederic Dapozze^b^, Aurélie Habert^a^, , Vincent Maurel^c^ and Nathalie Herlin-Boime^a*^

*^a^ NIMBE, CEA, CNRS, Université Paris Saclay, F-91191 Gif sur Yvette, France*

*^b^ Institut de recherche sur la catalyse et l’environnement, IRCELYON, CNRS-University of Lyon, Villeurbanne 69100, France*

*^c^ Univ. Grenoble Alpes, CEA, CNRS, INAC, SyMMES, F-38000 Grenoble, France*

*^d^ Laboratoire de chimie physique, UMR 8000-CNRS, Bât. 349, Université Paris Saclay, 91405 Orsay, France*

***Supplementary data***

***Figure S1:*** *X-Ray diffractograms of pure and modified TiO_2_ powders*

|  |  |
| --- | --- |

***Figure S2:*** *XPS spectra of TiO_2_, N-TiO_2_ and Au/N-TiO_2_ powders (a) Ti 2p and (b) O 1s*

In Au/TiO_2_ and Au/N-TiO_2_ samples, we expected the presence of metallic gold at 84.0 and 87.7 eV, it was not observed probably due to the low gold content.

Table S1 : Characteristics of pure and modified TiO_2_ photocatalysts

| Catalyst | Average size (TEM) (nm) | XRD size (nm) | Amount of Au « doping »  (%m ICP) | Amount of N « doping »  (%m ICP) |
| --- | --- | --- | --- | --- |
| P25  (TiO_2_ commercial) | 25 | 24 | 0 | 0 |
| TiO_2_ LP | 7.5 ± 1.4 | 7.9 | 0 | 0 |
| Au/TiO_2_ | 7.0 ± 1.6 | 7.0 | 0.16 | 0 |
| N-TiO_2_ | 9.0 ± 2.0 | 7.5 | 0 | < 0.05 |
| Au/N-TiO_2_ | 8.5 ± 1.8 | 8.5 | < 0.05 | < 0.05 |

**Figure S3:** Numerical simulation of N-TiO_2_ experimental EPR spectrum with no light

Dark curve experimental spectrum as reported in Figure 2 B. Red curve, numerical simulation of the EPR spectrum made by summing the EPR spectra of the following species (respective weights in brackets): NO^2-^ (23%), NO° (15%), Ti^3+^ with g tensor similar rutile C doped TiO_2_ (33%), electron in oxygen vacancy (2%) and surface Ti^3+^ with g tensor similar to water dispersed TiO_2_ at pH=10 (0,27%). See Table 1 for the EPR parameters of these species.

**Figure S4:** EPR spectra observed for dry ) Au/N-TiO_2_ sample powder without and with in situ UV-visible (halogen-lamp) irradiation at T=60K. (see exp. Section for details).
